# Protein biochemistry and engineering drive the development of a carbonic anhydrase‐based carbon dioxide sequestration strategy

**DOI:** 10.1111/febs.17416

**Published:** 2025-01-27

**Authors:** Loredano Pollegioni, Gianluca Molla

**Affiliations:** ^1^ ‘The Protein Factory 2.0’, Dipartimento di Biotecnologie e Scienze della Vita Università degli Studi dell'Insubria Varese Italy

**Keywords:** protein engineering, reaction mechanism, stabilization, structure–function relationships

## Abstract

The sequestration of carbon dioxide using carbonic anhydrase (CA) is one of the most effective methods for mitigating global warming. The burning of fossil fuels releases large quantities of flue gas; because of its high temperature and of the alkaline conditions required for CaCO_3_ precipitation in the mineralization process, thermo‐alkali‐stable CAs are needed. In this context, Manyumwa *et al*. conducted a biochemical characterization of three CAs derived from thermophilic bacteria. They then employed a rational design approach to enhance the specific activity and stability of the enzyme from the hydrothermal vent species *Persephonella* sp. *KM09‐Lau‐8*.

## Graphical abstract

CO₂ emissions reached unprecedented levels in 2024, significantly contributing to climate change. Carbonic anhydrases, such as the enzyme from *Persephonella* sp., offer a promising approach for CO₂ sequestration by converting it into bicarbonate. Engineering these enzymes to enhance stability and catalytic efficiency is crucial for their large‐scale application in industrial processes. This could lead to more effective bio‐based carbon capture technologies, addressing the urgent need to reduce atmospheric CO₂ levels.

Comment on: https://doi.org/10.1111/febs.17346.

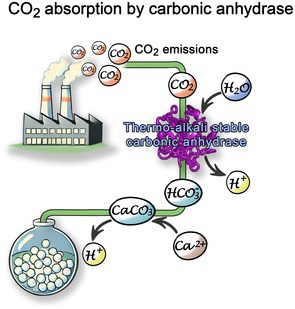

**Abbreviations**
CA: carbonic anhydrase
GHG: greenhouse gases
PhyCA: *Persephonella hydrogeniphila* carbonic anhydrase

## Introduction

Over the past 250 years, anthropogenic activities have caused a significant increase in greenhouse gases (GHG), including a ~ 35% rise in CO_2_ concentration in the atmosphere. Of this increase, two‐thirds is contributed by burning of fossil fuels. CO_2_ levels in the atmosphere have now surpassed the 400 ppm threshold, and they may remain above this level for generations. The increase in GHG emissions has led to a rise in Earth's surface temperature by about 1.5–2.0 °C compared with preindustrial times, contributing to natural calamities and negatively impacting the environment. Carbon sequestration is a process aimed at extracting significant amounts of GHG from the atmosphere and safely storing them elsewhere: By preventing CO_2_ from entering the atmosphere, carbon sequestration holds enormous potential for mitigating climate change. This process naturally occurs in Earth's ecosystems, such as grassland and forest plants, soils, and oceans, which act as natural CO_2_ sinks. However, scientists can also activate and enhance this process through current technologies to artificially capture CO_2_ emissions. Additionally, CO_2_ reuse is a cutting‐edge technology for the simultaneous reduction in atmospheric emissions and the production of raw materials by converting CO_2_ into other chemical compounds.

Carbon dioxide sequestration can be achieved through physical, chemical, and biological methods [1, 2, 3]. Physical methods involve adsorption onto porous materials or absorption into the liquid phase, though their removal efficiency tends to be relatively low; chemical methods rely on a chemical reaction between the absorbent and CO_2_ to generate high value chemicals (anyway the absorbents—typically strong alkaline compounds—can cause secondary environmental pollution); biological treatments utilize photoautotrophic organisms to convert CO_2_ into energy or aim to accelerate the CO_2_ adsorption rate into water. In the latter case, the enzyme carbonic anhydrase (CA, EC4.2.1.1), a zinc‐containing enzyme found in many organisms, is commonly used. CAs catalyze CO_2_ hydration reactions to form bicarbonate and protons (Eqn 1).

(1) $${\mathrm{CO}}_{2\left(g\right)}+H_{2}O_{\left(l\right)}\to H_{2}{\mathrm{CO}}_{3\left(\mathrm{aq}\right)}\to H_{\left(\mathrm{aq}\right)}^{+}+{\mathrm{HCO}}_{3\left(\mathrm{aq}\right)}^{-}$$

**Fig. 1:**
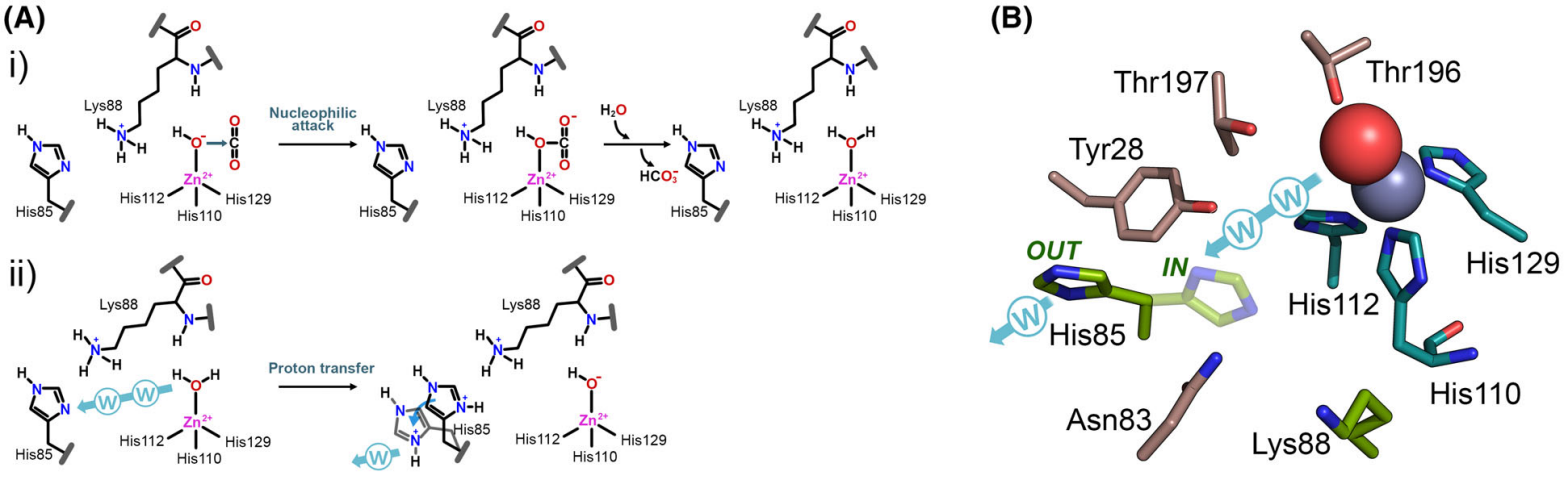
Catalytic mechanism and structure of PhyCA. (A) CO_2_ hydration reaction: (i) nucleophilic attack by the zinc‐bound hydroxyl ion to CO_2_; the produced ${\mathrm{HCO}}_{3}^{-}$ is displaced by a water molecule; (ii) proton transfer via a H‐bond network. Adapted from [4], which is copyrighted under a CC‐BY‐4.0 license. (B) Active site structure of the active site of PhyCA. The 3D model was built using alphafold3. The zinc (gray) and water (red) are represented as spheres. Residues coordinating zinc are in teal, residues involved in the proton shuttling H‐bond network are in brown, and residues investigated in the work from Manyumwa *et al*. [13] are in green (His85 is represented into two potential conformations). Direction of proton transfer is represented by a cyan arrow with W representing potential water molecules.

This reaction is composed of two half‐reactions (Fig. 1A) [4]. The first step involves the nucleophilic attack of a zinc‐bound hydroxide to a CO_2_ molecule, followed by the formation of bicarbonate coordinated to the metal ion; this bicarbonate is then quickly displaced by a water molecule, subsequently generating the acidic form of the enzyme, which is not catalytically active. The second step of the reaction is comprised of the regeneration of the zinc‐bound hydroxide through the transfer of a proton from the zinc‐bound water molecule to the external buffer.

CAs possess a very high catalytic activity (*k*_cat_), in the 10^5^–10^6^ s^−1^ range, and are thus used for enhancing CO_2_ hydration and precipitation to calcium carbonate (CaCO_3_). CA‐based reactors for capturing CO_2_ have been developed [5]: these processes require immobilized thermo‐alkali‐stable CAs since in postcombustion capture, the gas mixture is released at very high temperatures. For example, a process utilizes a highly thermostable engineered β–CA from *Desulfovibrio vulgaris* [6]: the rate of CO_2_ absorption increased by about 25‐fold in the catalyzed reaction as compared to the noncatalyzed one. Over the years, CAs from other sources have been used, such as the α‐class enzymes from *Caminibacter mediatlanticus* and *Sulfurihydrogenibium yellowstonense* YO3AOP1 [7, 8].

## Rational engineering of an enhanced carbonic anhydrase: production of an evolved enzyme for carbon dioxide sequestration

Protein engineering strategies to enhance enzyme performance for biotechnological applications typically aim to improve either catalytic activity or stability. To increase catalytic efficiency, these approaches often target residues involved in the rate‐limiting step of the catalytic cycle. For α‐CA, the rate‐limiting step involves the transfer of a proton from the zinc‐bound water molecule to the external solvent, which regenerates the zinc‐bound hydroxide (Fig. 1A). This proton transfer relies on a residue acting as a base (i.e., with a pK_a_ close to neutrality) to facilitate transfers of the proton from the catalytic water molecule to the bulk solvent through a well‐ordered H‐bonded water network [9]. Accordingly, substituting a catalytic lysine residue (pK_a_ = 8.6) with a histidine in human CA III increased the *k*_cat_ nearly 20‐fold [10]. In most α‐CAs, a histidine residue (e.g., His64) with a pK_a_ in the 6.25–7.60 range is typically present [11]. In bacterial α‐CAs, however, two basic residues (a histidine and a lysine) may participate in proton transfer; in CA from *Persephonella hydrogeniphila* (PhyCA), these residues are His85 and Lys88 (Fig. 1B).

A common assertion in protein engineering is that the combined impact of point mutations on enzyme activity and stability is often unpredictable. Enhanced activity frequently comes at the expense of reduced stability, as seen during the *in vitro* evolution of PET hydrolases [12]. However, substitutions at position Lys88 of PhyCA, introduced by Manyumwa *et al*. [13], produced variants with improved reaction rates and, in some cases, increased stability. For instance, the K88Q variant demonstrated a 10 °C increase in thermotolerance compared with wild‐type PhyCA. This enhancement appears characteristic of this class of bacterial enzymes. Recently, the same researchers engineered α‐CA variants from *Nitratiruptor tergarcus* that exhibited simultaneous improvements in both activity and stability [14]. Notably, the most effective variants involved substitutions at the enzyme's surface or dimerization interface. A combination of these substitutions produced a double variant (N88K/R210L) that retained 47% of its activity after 24 h of incubation at 90 °C [14].

In the study from Manyumwa *et al*. [13], the authors emphasized a distinctive feature of CAs: multiple residues at the active site can act as proton shuttles, albeit with differing efficiencies [15]. Interestingly, replacing the Lys88 proton shuttle side chain with alanine produced an enzyme with slightly increased catalytic activity. This finding aligns with studies on human α‐CA, where the removal of the conserved His64 residue at the active site yielded a variant retaining approximately 50% of the original activity [16].

Optimizing enzymes for biotechnological applications requires balancing improvements in both activity and stability, rather than prioritizing one property exclusively. This approach has been applied in designing thermostable bacterial α‐CAs by introducing novel disulfide bonds. For example, the double variant (N63C/P145C) exhibited reduced activity at 25 °C compared with the wild‐type enzyme but demonstrated thermo‐activation at elevated temperatures, retaining 56% of its activity after 24 h at 70 °C [17]. Applying this principle, Manyumwa *et al*. identified the PhyCA K88Y variant as the most promising candidate. While not the most active or stable variant overall, K88Y retained nearly 50% of its initial activity after 1 h at 90 °C, making it well‐suited for CO₂ sequestration under high‐temperature conditions.

## Conclusion

Until now, humans have not successfully removed atmospheric pollutants on a global, continental or regional scale. The only viable option has been to shut down the source and allow nature to restore balance. Carbon dioxide removal, however, presents a particularly challenging task, leaving us to mainly rely on the environment to stabilize atmospheric CO_2_ levels over time. CAs appear to be promising tools in addressing this issue, and biotechnological approaches are emerging as the most effective strategy. Advances in biotechnology are expected to enhance CO_2_ capture and sequestration processes. Bioinformatics tools can aid in discovering novel CA‐encoding genes and predicting beneficial substitutions. Additionally, enzyme engineering and immobilization techniques can improve CA activity and stability under operational conditions [6]. System biology approaches, along with the development of continuous operating reactors, will help tackle current challenges, ultimately paving the way for cost‐effective CO_2_ sequestration technologies.

## Conflict of interest

The authors declare no conflict of interest.

## Author contributions

LP and GM wrote the manuscript.
